# Soil pH and nitrate shape deterministic assembly of microbial communities in agricultural soils via Nitrososphaeria

**DOI:** 10.1128/aem.02067-25

**Published:** 2025-12-08

**Authors:** Huizhen Yan, Yunhua Zhang, Zhiguo Zhang, Ze Zhao, Lu Zhang, Feng Ju

**Affiliations:** 1 Research Center for Industries of the Future, Westlake Center of Synthetic Biology and Integrated Bioengineering, School of Engineering, Westlake University 726413, Hangzhou, Zhejiang Province, China; 2 Institute of Advanced Technology, Westlake Institute for Advanced Study, Hangzhou, Zhejiang Province, China; 3 Westlake Laboratory of Life Sciences and Biomedicine, Center for Infectious Disease Research, School of Life Sciences, Westlake University 572147, Hangzhou, Zhejiang Province, China; 4 Center for Future Foods, Muyuan Laboratory, Zhengzhou, Henan Province, China; University of Georgia Center for Food Safety, Griffin, Georgia, USA

**Keywords:** prokaryotic microorganism, agricultural soil, community assembly, Nitrososphaeria, ammonia-oxidizing archaea (AOA)

## Abstract

Understanding the assembly mechanisms of soil microbial communities is critical for maintaining nitrogen cycling in agricultural ecosystems, which underpins soil fertility and sustains crop productivity. While environmental filtering and biotic interactions shape these communities, our understanding of how functional taxa interact with soil properties across extensive agricultural landscapes remains limited. Here, we investigated the influence of environmental factors on the Chinese agricultural soil microbiome, integrating assessments of microbiota diversity, composition, and assembly process. The results indicated that soil pH and moisture were among the strongest abiotic factors explaining the agricultural soil microbiota compositional variation at a continental scale, surpassing the examined geographical and climatic effects. Stochastic processes dominated the assembly of microbial communities in large-scale agricultural soils, whereas the relative importance of deterministic processes increased with rising pH from acidic to alkaline soils. Phylogenetic turnover, as indicated by the beta nearest taxon index (βNTI), revealed determinism peaked under nitrogen-limited conditions but weakened with moderate precipitation, suggesting that both extreme aridity and rainfall amplify environmental filtering. We also found that divergent environmental preferences were displayed by ammonia-oxidizing microorganisms, including four archaeal genera belonging to the Nitrososphaeria class. Their significant correlations with βNTI as well as soil pH, nitrate, and moisture suggested that soil properties likely influenced prokaryotic community assembly primarily through modulating these functional taxa. This study highlights the vital role of ammonia-oxidizing-related soil properties in shaping the functional groups and assembly mechanisms of soil microbial communities, while enhancing our understanding of how ecological niche modifications by ammonia-oxidizers influence community interactions and nutrient dynamics in agricultural soils.

**IMPORTANCE:**

Agricultural soil microbiomes are essential for element cycling, fertility maintenance, and crop productivity, yet how key functional taxa interact with environmental factors to shape community assembly remains poorly understood. In this transcontinental study spanning diverse vegetation types, we demonstrate that ammonia-oxidizing archaea mediate soil microbial community assembly in response to pH and nitrate levels, with evidence of nonlinear threshold effects driven by nitrate. These findings underscore the pivotal role of keystone taxa in structuring soil biodiversity and ecological functions. Our study offers valuable insights into microbially mediated carbon and nitrogen cycling under climate change and supports crop-specific soil management strategies for sustainable agriculture.

## INTRODUCTION

In terrestrial ecosystems, soil microorganisms play a vital role in ecosystem health, environmental sustainability, and human well-being. They are essential for the biogeochemical cycles of earth’s elements (e.g., nitrogen, carbon, and phosphorus), support plant-associated microbial communities, shape the herbivore microbiome, and interact with many eukaryotes (1–4). The ecological function of soil microbiome has gained increasing recognition due to its profound implications for human, crop, environmental, and animal health under the One Health concept proposed in 2000 (5). To understand and predict the ecological function of the soil microbiome, it is essential to elucidate its community composition and assembly mechanisms. The microbial community is collectively shaped by both niche-based deterministic processes and neutral-based stochastic processes, and a major research focus lies in quantifying the relative importance of these two ecological processes. In specific types of farmland ecosystems, soil microbial taxa could be governed by distinct assembly processes and environmental factors (6, 7). Yet across heterogeneous agricultural landscapes spanning wider geographical extents and diverse vegetation types, the influence of these abiotic drivers varies as ecosystem complexity escalates (8).

Beyond their ecological significance, soil microorganisms are integral to agricultural productivity through their involvement in nutrient synthesis and transformation processes critical to soil biological and biochemical functioning (9, 10). However, the environmental drivers shaping soil microbial diversity in response to multifaceted environmental changes within agricultural systems remain poorly understood (11). Edaphic factors, climatic factors, and land-use intensification are the main impact factors on the soil microbiome (12–18). Among edaphic factors, including soil pH, temperature, organic carbon content, humidity, and spatiotemporal heterogeneity, are major drivers of soil microbial community diversity and assembly process, with feedback of their contributions to one health (12). The soil microbiome diversity and assembly process also change with climate fluctuation, such as global warming caused by greenhouse gas emissions (carbon dioxide) or climatic differences due to geographical patterns (19). Besides environmental factors, human farmland management also plays a key role in the health of soil microecology, which is reflected in chemical pollution (especially antibiotics and pesticide exposure) and land-use intensification (14, 19, 20). Although multiple environmental factors collectively shape soil microbiome composition, their relative contributions to microbial diversity and community assembly processes in Chinese agricultural soils remain lacking systematic assessment at a continental scale.

In agricultural soil, agricultural intensification and land use change were deemed to reduce microbial abundance and the overall diversity of soil organisms (21, 22). Among them, ammonia-oxidizing microorganisms, as key drivers of the nitrification process, can directly influence soil nitrogen cycling efficiency and crop nitrogen utilization through changes in their community structure (23), ultimately affecting the nitrogen supply capacity and crop productivity in agricultural systems. Hence, it is important to understand how changes in environmental factors influence soil biodiversity and, in particular, key functional taxa, and ultimately alter ecosystem functioning.

Particularly, China has a variety of climate zones, complex weather patterns, and diversified agricultural practices (24). This complexity underscores the unique challenges and opportunities for sustaining and enhancing agricultural productivity in China. Although numerous studies have shown that soil microbial community assembly depends on distinct environmental variables, most have been limited to individual or a narrow range of agricultural systems. A fundamental gap remains in understanding how key functional microbial taxa modulate assembly processes across diverse agroecosystems spanning complex environmental gradients. In this study, a large-scale soil survey was conducted between 13 and 25 April 2021 to investigate the diversity, composition, and assembly processes of soil prokaryotic communities across different vegetation types, sampling depths, and climate zones throughout China. A total of 205 soil samples were collected from 125 agricultural fields, with 9 edaphic variables measured. Through 16S rRNA gene amplicon sequencing with bacterial-archaeal universal primers, combined with null models and multivariate statistical analyses, this study aimed to test the overarching hypothesis: environmental factors exert priority effects on specific functional taxa, whose responses subsequently cascade to shape the assembly of the broader soil prokaryotic community. Specifically, we examined whether (i) edaphic properties acted as the primary drivers of microbial diversity and compositional variation, despite the wide geographical span of over 4,000 km; (ii) microbial assembly processes were expected to vary substantially along environmental gradients, such as pH and nitrate concentration; and (iii) as a key nitrogen-cycling archaeal taxon, Nitrososphaeria may mediate the integrated effects of edaphic, climatic, and geographic factors on soil microbial community assembly. This study would enhance our understanding of agricultural soil microbiota assembly mechanisms, especially the interaction between key functional taxa and abiotic drivers, highlighting the importance of biological nitrogen cycling processes in the soil microbiota assembly.

## MATERIALS AND METHODS

### Soil sampling, biogeochemical measurement, and data collection

**Fig 1:**
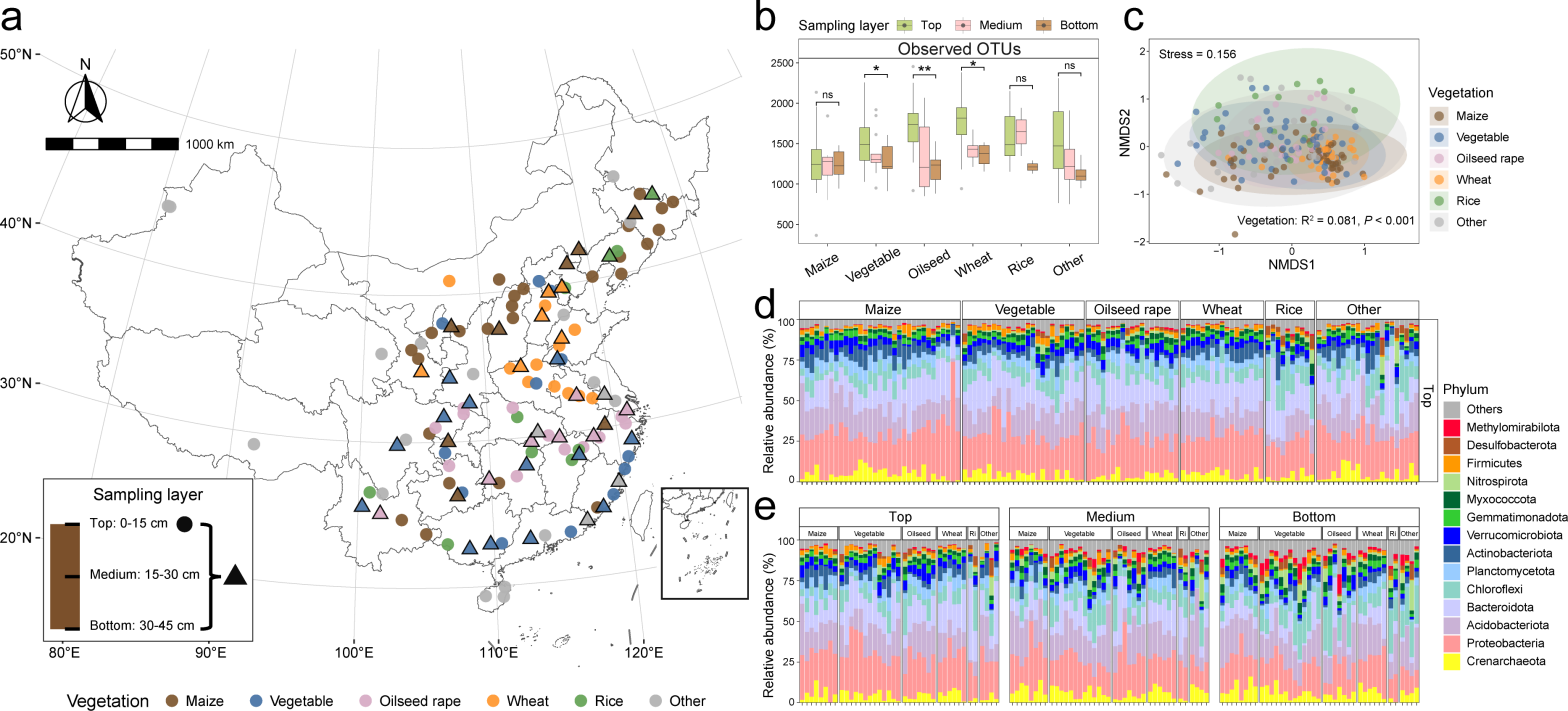
Biogeographical patterns of microbial community structure in China’s agricultural soils. (**a**) Location of 125 sampling sites investigated in this study. Site-specific vegetation types are shown by fill colors, with circles and triangles indicating surface-only and multi-depth sampling sites, respectively. The map was downloaded from the Tianditu platform under Map Review Approval Number: GS (2024) 0650, provided by the Ministry of Natural Resources of China. (**b**) Spatial variation in prokaryotic α-diversity across vegetation types and sampling layers (*n* = 205). Differences in α-diversity across sampling depths were tested by the Kruskal-Wallis rank sum test: ns = not significant; ^*^P < 0.05, ^**^P < 0.01. (**c**) Non-metric multidimensional scaling (NMDS) ordination based on Bray-Curtis dissimilarity, illustrating the compositional variation of microbial communities across vegetation types. (**d and e**) Relative abundances of dominant prokaryotic phyla (with average relative abundance > 1% across all samples) in (**d**) topsoil only and (**e**) three distinct soil layers, respectively.

Soil samples were collected from agricultural land across 31 provincial-level administrative regions of mainland China from 13–25 April 2021, including temperate, subtropical, and highland climate zones. The soil samples were transported and preserved until further processing as previously described (18). Briefly, a total of 205 soil samples were collected from 125 agricultural fields spanning 31 provincial-level administrative regions across China. These fields encompassed five dominant vegetation types (maize, vegetable, oilseed rape, wheat, and rice) across three distinct climate zones: subtropical, temperate, and high mountain (Fig. 1a; Fig. S1). We integrated the legacy data (*n* = 70) from Zhang et al. (18), which focused on bacterial secondary metabolites, with our additional new samples (*n* = 135). All 205 samples were collected, processed, and sequenced under the same conditions, an approach that substantially enhanced spatial coverage and statistical power for evaluating the environmental drivers of microbial community assembly. For each site, five soil cores obtained at a depth of 0–15 cm (top) were combined. Additionally, the samples were collected at depths of 15–30 cm (medium) and 30–45 cm (bottom) from 40 of the sampling sites (Data Set S1). Standard test methods were employed to measure soil pH, soil moisture (SM), dissolved organic carbon (DOC), total nitrogen (TN), nitrate nitrogen (NO_3_^−^-N), ammonium nitrogen (NH_4_^+^-N), available phosphorus (AP), and available potassium (AK), as previously described (18). Briefly, soil pH and AK were determined according to the protocols in the Agricultural Chemistry Committee of China. TN was determined using a Flash 2000 NC Analyzer (Thermo Scientific, MA, USA). The concentrations of NO_3_^−^-N and NH_4_^+^-N were assessed colorimetrically by automated segmented flow analysis (AAIII; Bran and Luebbe, Germany) using the salicylate/dichloroisocyanuric acid and cadmium column/sulfanilamide reduction methods, respectively. AP was extracted by 0.5 M NaHCO_3_ and determined using the molybdenum blue method. Climate data, including mean annual temperature (MAT) and mean annual precipitation (MAP) at a 2.5-min resolution, were obtained from the WorldClim database (www.worldclim.org).

### DNA extraction, PCR amplification, and 16S rRNA gene amplicon sequencing

For each soil sample, DNA was extracted using the FastDNA Spin Kit for Soil (MP Biomedicals, USA), then its purity and concentration were assessed using a Nanodrop One spectrophotometer (Thermo Fisher Scientific, MA, USA). The V4-V5 hypervariable regions of prokaryotic 16S rRNA genes were amplified using the forward primer 515F (5′-GTGYCAGCMGCCGCGGTAA-3′) and reverse primer 926R (5′-CCGYCAATTYMTTTRAGTTT-3′) as recommended by the Earth Microbiome Project (https://earthmicrobiome.org/) (25). The amplicons from each DNA sample were evenly mixed and then sequenced on the Illumina NovaSeq 6000 platform (PE250) at the Magigene Biotechnology Corporation (Guangzhou, China).

### Bioinformatics analysis of high-throughput sequencing data

The amplicon sequencing data were then processed in QIIME2 (26), as described in our previous study (27). Briefly, the DADA2 plugin was used to merge, quality-filter, trim, denoise, and remove chimeras from the raw paired-end reads, generating amplicon sequence variants (ASVs). The representative ASV sequences were subsequently clustered at 97% similarity using the VSEARCH plugin to obtain OTUs for downstream analyses. The OTUs were taxonomically assigned using a Naïve Bayes classifier against the SILVA rRNA database (version 138). Chloroplast and mitochondrial sequences were removed, along with any sequences that could not be assigned to bacteria or archaea. Alpha diversity (including richness, Shannon-Wiener index, and Faith’s phylogenetic diversity) metrics were calculated using the QIIME 2 “diversity” plugin based on an OTU table rarefied to an even depth of 33,130 reads per sample. Taxonomic abundance profiles at the phylum and genus levels were generated using the “taxa” plugin.

### Microbial community assembly mechanism analysis

Microbial community assembly patterns and processes were systematically analyzed using MbioAssy2.0 (https://github.com/emblab-westlake/MbioAssy) (28), which implements the “qpen” function in “iCAMP” to quantify the relative contribution of deterministic (selection) and stochastic (dispersal, drift, and diversification) processes in community assembly (29). Meanwhile, the beta nearest taxon index (βNTI) was used to quantify phylogenetic turnover between each pairwise comparison. A βNTI value less than −2 indicates significantly lower phylogenetic turnover than expected by chance, suggesting homogeneous selection, whereas a βNTI value greater than +2 indicates significantly higher phylogenetic turnover than expected, implying variable selection (30, 31). A |βNTI| < 2 indicates the dominance of stochastic processes.

### Multivariate statistical analysis

The Bray-Curtis dissimilarities in microbiota composition between soil samples were calculated using the “vegan” package, followed by non-metric multidimensional scaling analysis with the “metaMDS” function and distance-based redundancy analysis (dbRDA) with the “capscale” function. Permutational multivariate analysis of variance (PERMANOVA) was performed using the “adonis” function in the “vegan” package to test the significance of community compositional variation across vegetation types and sampling depths, and climate zones. The Kruskal-Wallis rank sum test was used to test the significance of variations in community alpha diversity among different vegetation types and sampling depths. Spearman’s rank correlations between environmental factors and both microbial community diversity and dominant taxon abundances were computed using the “corr.test” function from the “psych” package. Furthermore, environmental factors that influenced the assembly processes of soil microbiota were investigated. Variation in community assembly processes along the gradients of the environmental factors was assessed using the Mantel test which correlated the βNTI values with the Euclidean distance matrices of each factor. Breakpoint analysis of the relationship between βNTI and nitrate Euclidean distance was conducted using the “segmented” function in the “segmented” package. The significance of differences in nitrate concentration and prokaryotic community βNTI among groups ranked by environmental factors was assessed using the “kruskal.test” function in the “stats” package.

The relative importance of the geographic, climatic, and edaphic variables on the soil microbial community dissimilarities was determined based on variation partitioning analysis (VPA) using the “varpart” function in the “vegan” package. Distance-decay relationships (DDRs) were evaluated by linear regression between pairwise Bray-Curtis similarities of community composition and geographic distances, with the latter calculated using the “distm” function in the “geosphere” package. Surface soil samples were clustered using hierarchical clustering with complete linkage via the “pheatmap” package, and subsequently divided into low, medium, and high sub-groups. Besides, Partial Least Squares Path Modeling (PLS-PM) analysis was used to explore the direct and indirect effects of geographic factors (*Latitude*; longitude was excluded from the final model due to its negligible loading), climatic factors (*Climate*; including MAP and MAT), *pH*, inorganic nutrients (*Nutrients*; including NO_3_^−^-N and AP as identified by the prior Mantel test), Nitrososphaeria (*AOA*) abundance and composition (represented by PCoA1 axis scores based on the Bray-Curtis dissimilarities) on microbial community assembly (captured by the PCoA1 axis scores derived from the βNTI matrix). In brief, a hypothesized model that included all reasonable pathways. Nonsignificant pathways were sequentially pruned, or new pathways were added based on residual correlations, until the model showed sufficient fitting. The PLS-PM analysis was performed using the “plspm” package. OTUs with an average relative abundance ≥0.05% and present in at least 25% of the samples were selected for co-occurrence network analysis using the ‘rcorr’ function from the “Hmisc” package. Only associations between Nitrososphaeria OTUs and other taxa with a correlation coefficient (r) ≥ 0.5 and a *P*-value < 0.05 were visualized using Cytoscape v3.10.3.

## RESULTS

### Microbial diversity and composition patterns in agricultural soils

To investigate the composition and diversity of the agricultural soil microbiome across China, a total of 205 soil samples were collected from six vegetation types, three soil depths, and three climate zones (Fig. 1a; Fig. S1). The full prokaryotic data set (*n* = 205) yielded 29,899 OTUs comprising 15,251,281 clean reads (ranged 33,132–168,632, mean = 74,396 per sample). The sequencing depth in this data set adequately captured the overall species diversity of soil prokaryotic communities and Nitrososphaeria (Fig. S2). In surface soils, the α-diversity of prokaryotic communities varies significantly across vegetation types (*P* < 0.001, *n* = 125), whereas no significant differences are observed across vegetation types in the middle (15–30 cm, *P* = 0.750, *n* = 40) and deep (30–45 cm, *P* = 0.423, *n* = 40) soil layers (Fig. 1b; Fig. S3a; Table S1). The richness of microbial species (as proxied by OTUs) showed a decreasing trend with increasing sampling depth, most notably in vegetable, oilseed rape, and wheat fields. The soil microbial communities were dominated by Proteobacteria (6.15%–73.96%), Acidobacteriota (1.50%–47.76%), Bacteroidota (0.45%–50.24%), Chloroflexi (0.51%–39.11%), and Crenarchaeota (0.07%–16.06%) (Fig. 1d and e). Notably, Crenarchaeota was the only archaeal phylum with an average relative abundance greater than 1%, whereas 13 bacterial phyla exceeded this threshold. Most members within this archaeal phylum were assigned to Nitrososphaeria and Bathyarchaeia. PERMANOVA analysis showed that the geographic region explained the largest composition variations in the soil microbiota (R^2^ = 0.313, *P* < 0.001, *n* = 205), suggesting that human activities and management practices have a substantial impact on agricultural soil microbial communities. Regarding natural environmental factors, the results showed that vegetation type had the strongest effect on community composition, followed by sampling depth and climate zone (R^2^ = 0.042 to 0.81, all *P*-values < 0.001, Fig. 1c; Fig. S3b). These results indicated that China’s agricultural soil microbiome was geographically and vegetatively separated, which implied that the structure of the agricultural soil microbiome is shaped by both environmental factors and human activities.

### Drivers of variation in the composition of agricultural soil microbiota at a continental scale

**Fig 2:**
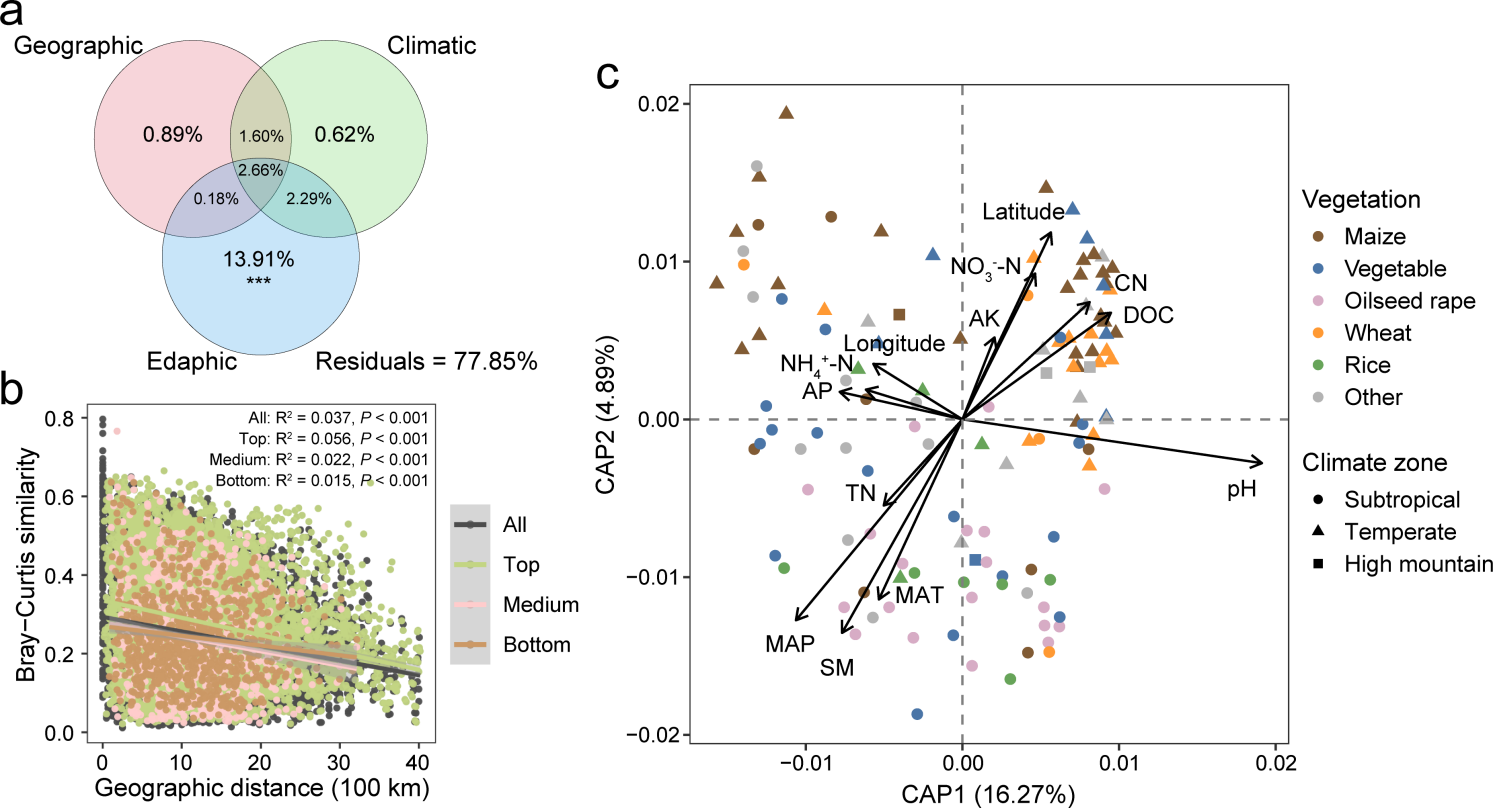
The influence of environmental factors on the soil microbial community composition. (**a**) VPA of the microbial community composition across geographic, climatic, and edaphic variables. The variation uniquely explained by each set of variables was determined after controlling for the effects of the others, based on ANOVA permutation tests (^***^P < 0.001). (**b**) DDR of soil microbial communities, with solid lines denoting significant linear regressions. (**c**) dbRDA of microbial communities with normalized environmental variables.

The relative contributions of edaphic, geographic, and climatic variables to explaining compositional variation were assessed using VPA (Fig. 2). The result showed that the complete set of all variables together explained 22.15% of the variation in microbiota composition, with soil edaphic properties contributing the most significantly (Fig. 2a). Geographic and climatic factors exerted comparable influences on community variation (0.89% and 0.62%, respectively), with the former showing a slightly stronger effect. Across all soil sampling depths, prokaryotic community similarity consistently decreased with increasing geographic distance between sampling sites, exhibiting clear and significant distance-decay patterns (Fig. 2b, all *P*-values < 0.001). Next, we explored the key environmental variables that shaped the soil microbial communities. The dbRDA revealed soil pH, MAP, and SM as the principal drivers shaping microbiota composition (Fig. 2c; Table S2). Among soil nutrients, DOC and nitrate (NO_3_^−^-N) exhibited the strongest correlations with community structure. Although α-diversity remained unresponsive to environmental gradients, pH emerged as a pivotal factor, significantly modulating the abundance of dominant phyla such as Crenarchaeota, Verrucomicrobiota, and Proteobacteria (Fig. S4). MAP, MAT, and SM displayed a consistent correlation pattern with these taxa, whereas DOC and NO_3_^−^-N acted in concert with pH, each showing significant positive associations with Crenarchaeota and Actinobacteria, respectively (Fig. S4). Notably, ammonium (NH_4_^+^-N) and latitude also exerted distinct selective pressures, influencing both microbial diversity and compositional profiles.

### pH and nitrate as key factors regulating microbial community assembly processes in the agricultural soils

**Fig 3:**
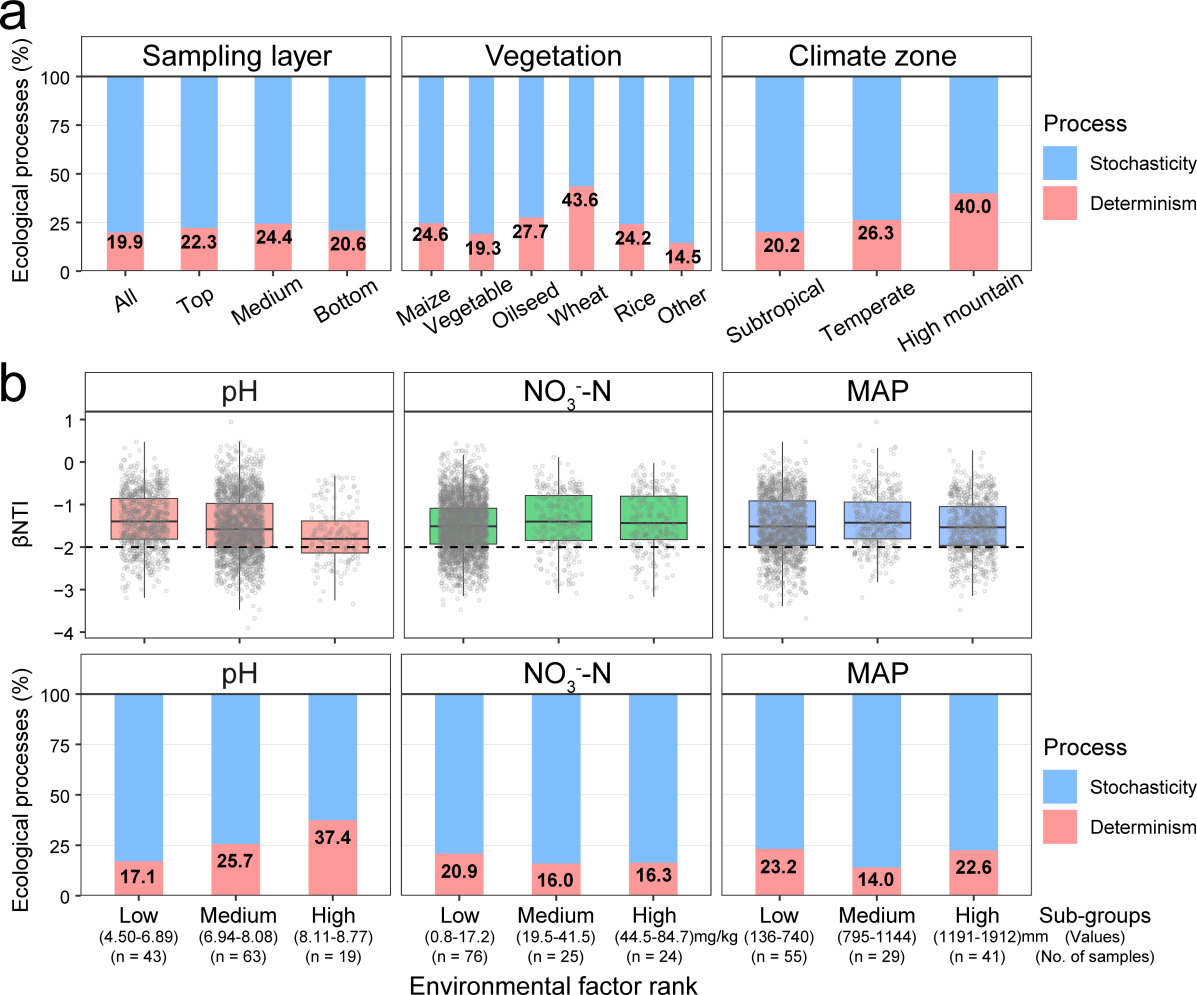
Assembly mechanisms of agricultural soil microbial communities. (**a**) Relative contribution of assembly processes among sampling layers, vegetation types, and climate zones using null models. (**b**) Patterns of βNTI across different categories in soil pH, NO_3_^−^-N, and MAP.

The pairwise βNTI statistical results across all samples indicated that soil prokaryotic communities at the continental scale were predominantly governed by stochastic processes, which account for 56.4%–85.5% influence. The relative influence of deterministic processes varies mostly with vegetation type (CV = 38.7%, coefficient of variation), followed by climate zone (CV = 35.1%), while showing minimal variation with sampling depth (CV = 8.5%) (Fig. 3a). Notably, among the vegetation types investigated, determinism exerted the strongest influence on soil prokaryotic communities in wheat fields, while its impact was weakest in vegetable fields. In the arid environment, the proportion of deterministic processes shaping maize soil microbial communities was much lower than that in wheat soils but was close to that in waterlogged and anaerobic rice paddy soils. A monotonic pattern was observed across climate zones, with determinism being most pronounced in high mountain zones and least evident in subtropical zones.

The relationships between microbial βNTI and differences in environmental variables were used to infer the correlations between community assembly processes and environmental factors. We found that pH and MAP were the primary factors influencing prokaryotic community assembly in wheat soils. In maize soils, significant drivers included pH, AP, and AK, whereas AK and DOC emerged as the key determinants in anaerobic rice paddy soils (Table S3; Fig. S5). Mantel test showed that soil pH (ρ = 0.253, *P* = 0.001), nitrate (ρ = 0.111, *P* = 0.009), and MAP (ρ = 0.103, *P* = 0.002) were the best predictors of assembly processes of the surface soil microbial community, irrespective of crop diversity, at continental geographic scales. (Table 1). Furthermore, the surface soil samples were categorized into low, medium, and high sub-groups based on the clustering results of pH (4.50−8.77), NO_3_^−^-N (0.8−84.7 mg/kg), and MAP (136−1,912 mm) values, and community assembly processes were subsequently quantified for each sub-group to evaluate the variation in determinism along environmental gradients (Fig. 3b). A total of 125 surface samples were classified into 43 low-pH, 63 medium-pH, and 19 high-pH sub-groups; 76 low-NO_3_^−^-N, 25 medium NO_3_^−^-N, and 24 high-NO_3_^−^-N sub-groups; and 55 low-MAP, 29 medium-MAP, and 41 high-MAP sub-groups. We then assessed the relative importance of assembly processes in each sub-group, although stochastic processes consistently overwhelmed deterministic ones in all sub-groups, deterministic processes showed apparent changes along pH, NO_3_^−^-N, and MAP gradients. With increasing soil pH, the relative importance of deterministic processes progressively increased. Microbial communities in agricultural soils exhibited the strongest deterministic assembly under alkaline conditions, whereas they showed the weakest deterministic patterns in acidic soils. When soil NO_3_^−^-N concentrations were below 17.2 mg/kg, deterministic processes exerted the strongest influence on prokaryotic communities, accounting for 20.9% of community assembly. However, when NO_3_^−^-N concentrations exceeded 19.5 mg/kg, the relative importance of deterministic processes declined to 16.0%. The βNTI of soil microbial communities under low nitrate concentrations differed significantly from those under nitrate-non-limiting conditions (Fig. S6). In agricultural soils with moderate levels of MAP, deterministic processes exerted the weakest influence on prokaryotic communities, whereas under conditions of low or high MAP, their influence was considerably stronger. These results strongly indicated that soil pH, nitrate nitrogen, and MAP are the primary drivers of assembly processes in agricultural soil microbial communities across China.

**TABLE 1:** Mantel test analysis of the relationship between surface microbial community βNTI and environmental factors^*a*^

Factor	Spearman		Pearson	
	ρ	*P*	R	*P*
pH	**0.253**	0.001^**^	**0.278**	0.001^**^
NO_3_^−^-N	**0.111**	0.009^**^	**0.107**	0.030^*^
MAP	**0.103**	0.002^**^	**0.111**	0.003^**^
AP	**0.091**	0.028^*^	0.084	0.056
Latitude	**0.087**	0.005^**^	**0.083**	0.022^*^
AK	0.079	0.053	**0.116**	0.031^*^
Longitude	**0.073**	0.042^*^	**0.091**	0.044^*^
MAT	**0.066**	0.048^*^	0.058	0.085
NH_4_^+^-N	0.053	0.111	0.031	0.226
SM	0.043	0.153	0.047	0.133
CN	0.0001	0.484	0.032	0.293
DOC	−0.006	0.531	0.007	0.430
TN	−0.071	0.946	−0.063	0.891

^*a*^ Significant correlation coefficients and *P*-values are in bold*. P*-values are marked with asterisks to indicate significance levels (^*^P < 0.05, ^**^P < 0.01; *n* = 125). CN, carbon to nitrogen ratio (dissolved organic carbon/total nitrogen); DOC, dissolved organic carbon; TN, total nitrogen; NO_3_^−^-N, nitrate nitrogen; NH_4_^+^-N, ammonia nitrogen; AK, available potassium; AP, available phosphorus; SM, soil moisture; MAT, mean annual temperature; MAP, mean annual precipitation.

### Nitrososphaeria mediates the influence of key factors on soil microbial community assembly processes

**Fig 4:**
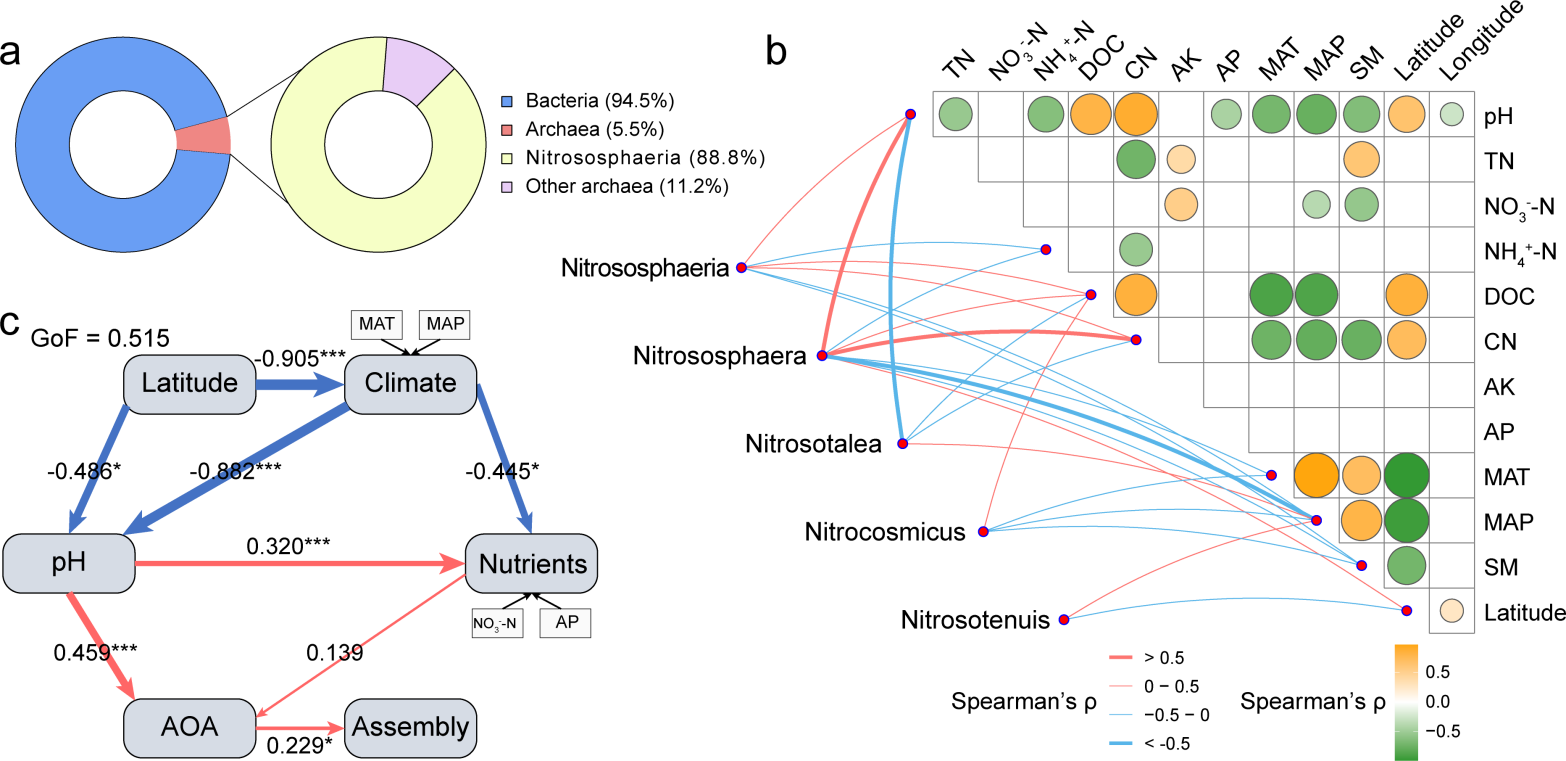
Nitrososphaeria as a mediator between environmental factors and microbial community assembly. (**a**) Proportion of Nitrososphaeria in archaea; (**b**) Heatmap of significant environmental factor correlations, with connecting lines indicating significant associations between Nitrososphaeria and environmental factors (Spearman’s ρ, *P* < 0.05). (**c**) PLS-PM describing the direct effects (path coefficients) on microbial community assembly processes. Numbers adjacent to arrows indicate effect sizes, with red and blue arrows denoting significant positive and negative paths, respectively. Only paths with *P* < 0.1 are shown. Path coefficients are annotated with asterisks to denote significance levels: ^*^P < 0.05, ^**^P < 0.01, ^***^P < 0.001.

The strong correlation between the concentration of nitrate nitrogen and microbial community assembly processes in large-scale agricultural soils across China suggests a critical role of microbes involved in nitrogen cycling in microbial community assembly despite their normally low abundance (<1%) in the soil (32, 33). To test this hypothesis, we assessed the relationships between nitrogen cycle-related microbes and community assembly processes. We noted that an archaea class, Nitrososphaeria, dominated in soil archaeal community (88.8%, Fig. 4a). The relative abundance of Nitrososphaeria (ammonia-oxidizing archaea [AOA]) ranged from 0.07% to 16.01%, with a mean of 4.93%, surpassing that of the classic ammonia-oxidizing bacteria (*Nitrospiria*), which ranged from 0.04% to 4.78% (mean: 1.16%). Nitrososphaeria played a significant role in shaping soil microbial community assembly (Mantel r = 0.349, *P* < 0.001); specifically, as their relative abundance within the community increased, the influence of deterministic processes on community assembly became more pronounced (Fig. S7). This archaeal class has been previously reported to participate in nitrification in geochemical processes (34). We then evaluated the correlations between the abundance of Nitrososphaeria and abiotic factors, with a focus on soil pH, nitrogenous nutrients, and MAP, which the Mantel test identified as the primary drivers of community assembly (Fig. 4b). We found that Nitrososphaeria showed significant positive correlations with pH (ρ = 0.480, *P* < 0.001), NO_3_^−^-N (Spearman’s ρ = 0.268, *P* = 0.130; Pearson’s r = 0.306, *P* = 0.033), and DOC (ρ = 0.397, *P* < 0.001), while exhibiting significant negative correlations with NH_4_^+^-N (ρ = −0.355, *P* = 0.003), MAP (ρ = −0.371, *P* = 0.001), and SM (ρ = −0.315, *P* = 0.020). Within the class Nitrososphaeria, the most abundant genus, *Nitrososphaera*, exhibited a significant positive correlation with pH, but a negative correlation with NH_4_^+^-N and MAP. In contrast, the second most abundant genus, *Nitrosotalea*, displayed an opposite pattern of correlations with these environmental factors. Additionally, other dominant genera within Nitrososphaeria, such as *Nitrocosmicus* and *Nitrosotenuis*, were primarily associated with DOC and MAP.

Subsequently, we performed PLS-PM analysis to assess the effects of geographic factors, climatic factors, soil physicochemical properties, and key functional groups on the assembly of soil microbial communities (Fig. 4c). The results showed that Nitrososphaeria (AOA) had a significant direct impact (path coefficients = 0.229, *P* = 0.043) on the assembly of soil microbial communities. Soil pH and NO_3_^−^-N indirectly influenced community assembly by affecting Nitrososphaeria. Climatic factors, including MAT and MAP, as well as latitude, primarily regulated the assembly of soil microbial communities indirectly by altering soil pH and NO_3_^−^-N levels. Given the model’s low goodness-of-fit, the presence of other unmeasured factors or complex nonlinear relationships cannot be ruled out and warrants further investigation. The co-occurrence network revealed that Nitrososphaeria OTUs were generally positively correlated with dominant bacterial taxa, with functional differentiation observed among different genera within Nitrososphaeria. Specifically, OTU28 (affiliated with *Nitrososphaera*) showed strong associations with OTUs from Planctomycetota, Alphaproteobacteria, and Gemmatimonadota; OTU20 (affiliated with *Nitrosotalea*) was closely linked to Acidobacteriota OTUs and OTU19 (Alphaproteobacteria); and OTU27 (affiliated with *Nitrocosmicus*) exhibited a close relationship with OTU62 (Alphaproteobacteria) and OTU105 (Acidobacteriota). These findings support our hypothesis that Nitrososphaeria are highly sensitive to variations in soil pH, nitrate, and precipitation and play a central mediating role in the assembly of soil microbial communities.

## DISCUSSION

As highlighted in *The Soil Microbiome: A Game Changer for Food and Agriculture* by FAO (35), disentangling key deterministic factors of microbial community assembly processes is vital for guided ecosystem management (36), particularly as agricultural soil microbiomes confront intensifying global environmental perturbations (37, 38). Thus, it is crucial to unveil the attributes of the agricultural soil microbial community and identify the primary factors influencing the assembly processes. This will offer anticipatory insights into the evolving challenges posed by environmental shifts, human activities, and intensified land use in the context of modern agriculture. In this study, we show on a continental scale (i) that soil microbiota exhibits distinct biogeographic and ecological patterns in diversity and composition, (ii) that soil properties, particularly pH and nitrate concentration, serve as the predominant drivers governing soil microbial community assembly, and (iii) that environmental preferences of Nitrososphaeria may interact with soil nitrate and moisture to regulate the assembly process of soil microbiota in agricultural fields.

The DDR depicts the decrease in community similarity as geographic distance increases, thus providing a directional model for changes in beta diversity across spatial scales (39). We observed only weak DDR (*R^2^* ≤0.056) across all sampling depths in China’s agricultural soil microbiota. The fitness values of these relationships were much lower than those reported in natural terrestrial ecosystems (40, 41), suggesting that human agricultural management may have disrupted the geographic pattern of soil microbiome in different regions. Our findings also revealed a parallel phenomenon where soil microbial diversity is influenced by a combination of geographic patterns, climate factors, and soil properties with previous studies (42–44). Notably, soil properties emerge as the predominant factor, playing the most critical role in shaping microbial diversity (7). Previous research has described edaphic properties, including soil pH, organic carbon, and nutrient composition, as important factors driving the homeostasis of soil microbial communities (45–48). Since soil microbes often interact with soil properties, the relationship between agricultural soil properties and microbial diversity needs to be further clarified by replicate experiments on control variables that can also resolve anthropogenic impacts (e.g., pollution) on soils.

**Fig 5:**
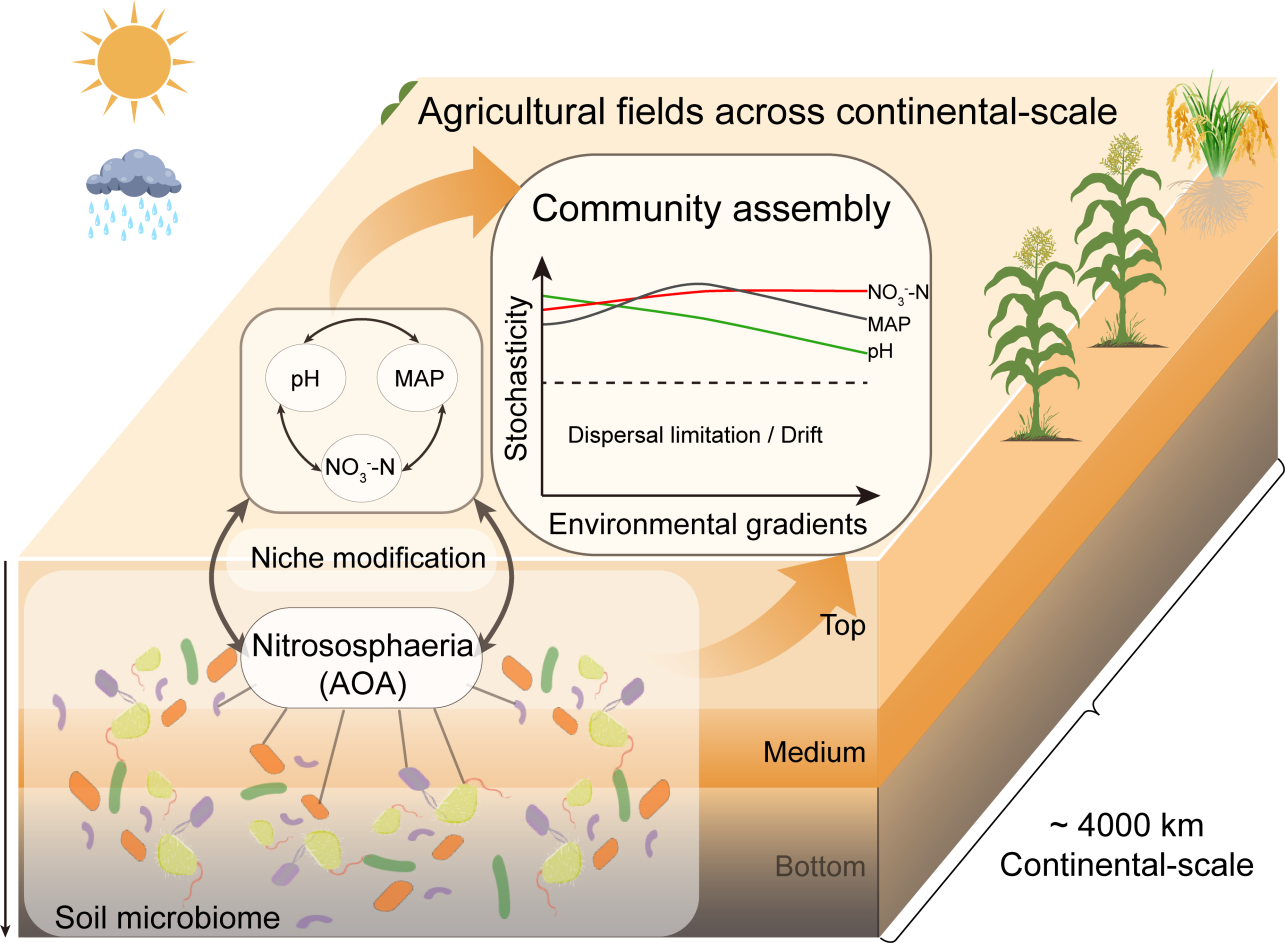
Conceptual model showing assembly patterns in microbial community along environmental gradients. Stochastic processes govern the assembly of large-scale agricultural soil microbial communities, although key factors collectively impose selection by influencing Nitrososphaeria. The contribution of stochasticity decreases with increasing pH, while along the nitrate gradient, it first rises and then levels off.

Consistent with our findings, a sibling study also demonstrated that soil pH and annual average temperature predominantly mediate the assembly of abundant and rare sub-communities, respectively, in agricultural fields of China (6). However, in this study, we did not distinguish between high abundance and rare microbial species. We found that the most important factors driving the assembly of soil microbiota were soil pH, nitrate, and MAP. Interestingly, the responses of soil prokaryotic community structure and function to dry-wet cycles were more stable in paddy than in dryland ecosystems (49). Our findings suggest that SM and MAP play a key role in soil microbial diversity and community structure. Previous studies (50, 51) indicate that heterotrophic respiration declines with SM, nearly ceasing under dry conditions, which underscores water availability as a primary constraint on microbial processes in well-drained soils. In agricultural systems, SM is largely influenced by irrigation practices and regional precipitation; therefore, these factors are critical in regulating microbial activity and community assembly. In addition, soil pH, nitrate, and precipitation were identified as key drivers shaping microbial community assembly in this study (Fig. 5). These key factors may exert combined effects on Nitrososphaeria, thereby regulating the assembly mechanisms of soil microbial communities. Determinism exhibits distinct patterns of variation along the gradients of these factors. Specifically, soil pH strongly governs community assembly processes globally, with extreme pH conditions imposing more stringent limitations on microbial survival, thereby exerting intensified selection pressures (52). Under nitrate nitrogen-limited conditions (<17.2 mg/kg), the relative importance of deterministic processes increased markedly, indicating nitrogen availability could enhance the influence of deterministic processes for agricultural soil microbiomes. The soil nitrate pool in these ecosystems originates mainly from anthropogenic fertilizer inputs and microbial nitrification, implying nitrate concentrations may restructure microbial communities through altered interaction networks (53). According to the results of China’s national soil survey, available nitrogen content in soil below 50 mg/kg is insufficient to meet the growth requirements of most crops. Concentrations below this range often indicate nitrogen deficiency, which can limit plant growth and indirectly exert stronger selective pressures on soil microbial communities. Conversely, empirical studies have shown that excessive nitrate accumulation in agricultural soils is linked to pollution of adjacent aquatic systems (54). As nitrate levels continued to rise, the contribution of deterministic assembly remained relatively constant, suggesting a nonlinear threshold response of nitrate availability on microbial community assembly. This indicates that once nitrate availability surpasses a certain threshold, its influence reaches an upper limit, and deterministic processes become shaped by other factors. Beyond this threshold, microbial communities tend to develop resistance to further changes in nitrate levels. This finding suggests that agricultural practices such as fertilization should be carefully managed to avoid surpassing this biological threshold, thereby preserving microbial diversity and maintaining key ecological functions.

Consistent with previous findings that extreme climatic events can profoundly alter soil microbial community functions and consequently threaten the stability of surface biospheres (55), our study similarly demonstrates that both excessive and insufficient precipitation enhance the role of deterministic processes in soil microbial community assembly. Precipitation may affect microbial community assembly by modulating SM. SM was positively correlated with precipitation, but negatively correlated with both pH and nitrate concentration. One plausible explanation supported by a rigorously controlled microcosm experiment (56) is that low water content accumulation promotes some nitrification-related microorganisms, leading to nitrate accumulation. Impressively, compared with dryland soils, waterlogged paddy soils provide a unique habitat due to the oxygen-limited conditions generated there during frequent flooding events (57). However, precipitation and long-term irrigation treatment of paddy fields may promote random processes inherent in agricultural soil, such as drift and dispersal limitations. As observed in this study, drought-tolerant crops such as wheat and rapeseed exhibited significantly stronger deterministic processes in soil microbial community assembly compared to vegetables and rice. One possible explanation is that water deficit intensifies nutritional limitations for soil microorganisms. Alternatively, distinct root exudate profiles associated with different crop types may selectively recruit specific microbial taxa (58), leading to priority effects that shape localized soil microbiomes. Overall, these findings suggest that under global change scenarios, policymaking and soil management should consider different agricultural systems, geographical patterns, and climate distribution to improve ecosystem productivity and sustainability.

Our findings indicate a substantial correlation between the archaea class Nitrososphaeria and nitrate, suggesting that Nitrososphaeria is a key taxon in maintaining agricultural soil fertility, playing important roles in CO_2_ fixation and the emission of greenhouse gases such as N_2_O (59, 60). Nitrososphaeria constitute a central component of nitrogen cycling in highly fertile agricultural soils (61). Under varying substrate and temperature conditions, they play a key role in determining nitrification potential and rates (62, 63), suggesting that they may influence the assembly of soil microbial communities by regulating nitrification. This correlation may elucidate why *Nitrososphaera* content is notably and negatively correlated with MAP and SM, suggesting that prolonged precipitation and irrigation could contribute to shaping anoxic geological conditions. Furthermore, environmental preference differences were also identified among Nitrososphaeria, for example, *Nitrososphaera* and *Nitrosotalea* preferred high-pH and low-pH environments, respectively, which is also supported by prior evidence that these two genera are correspondingly alkaliphilic and acidophilic archaeal ammonia oxidizers (64, 65). These consistent lines of evidence from other investigations strengthen the overall reliability of our predicted environmental preferences for these dominant AOA taxa. However, it is worth noting that the primer bias inherent in the bacterial-archaeal universal primers used in this study may have limited the detection of certain AOA species, potentially leading to an underestimation of the diversity within this group. Considering the distinct environmental preferences and potential functional differences among Nitrososphaeria genera, future studies should employ AOA-specific primers for sequencing to more reliably validate, with higher resolution, the pivotal role of this functional group in shaping microbial community assembly in agricultural soils.

Soil microbiome faces a myriad of compounding challenges, the effects of which may have widespread functional consequences above and below ground (66, 67). Our investigation of agricultural ecosystems revealed distinct environmental preferences of archaea, Nitrososphaeria, and demonstrated their associations with the agricultural soil nitrogen cycling and microbial assembly processes. These findings suggested that human management practices (e.g., farming or irrigation) in agricultural fields in China can strongly influence the relationship between community assembly and environmental factors under disturbed conditions. This study represents a considerable advance in linking climate, nitrogen cycle, and microbial community assembly. However, due to the uncontrollable variability of fertilization and irrigation management in the field, further research with controlled variables is needed to explore deeper internal interactions. Overall, this study provides a potential predictor of the response of agroecosystems to ongoing environmental changes, offering valuable insights for enhancing agricultural sustainability and improving crop productivity.

## Data Availability

The sequence data were deposited in the China National GeneBank (CNGB) database with the accession number CNP0005312. The main scripts used for sequence processing, statistical analysis, and visualization in this study have been deposited in the GitHub repository (https://github.com/huizhen-yan/Soil-microbiome).

## SUPPLEMENTAL MATERIAL

Data Set S1 aem.02067-25-s0001.xlsx Sampling information.

Supplemental material aem.02067-25-s0002.pdf Figures S1 to S8; Tables S1 to S3.

ASM does not own the copyrights to Supplemental Material that may be linked to, or accessed through, an article. The authors have granted ASM a non-exclusive, world-wide license to publish the Supplemental Material files. Please contact the corresponding author directly for reuse.
