# Supplementary material for: Soil pH and nitrate shape deterministic assembly of microbial communities in agricultural soils via Nitrososphaeria
Source: Appl Environ Microbiol. 2025 Dec 8;92(1):e02067-25. doi: 10.1128/aem.02067-25 (PMC12838428; doi:10.1128/aem.02067-25)
Supplement: Supplemental material — Figures S1 to S8; Tables S1 to S3. [file aem.02067-25-s0002.pdf]

SUPPLEMENTARY MATERIAL for

Soil pH and nitrate shape deterministic assembly of microbial communities in agricultural soils via Nitrososphaeria

Huizhen Yan[1,2#], Yunhua Zhang[1,2#], Zhiguo Zhang[1,2], Ze Zhao[1,2], Lu Zhang[1,2], Feng Ju [1,2,3,4*]

[1] Research Center for Industries of the Future, Westlake Center of Synthetic Biology and Integrated Bioengineering, School of Engineering, Westlake University, Hangzhou 310030, Zhejiang Province, China

[2] Institute of Advanced Technology, Westlake Institute for Advanced Study, Hangzhou 310024, Zhejiang Province, China.

[3] Westlake Laboratory of Life Sciences and Biomedicine, Center for Infectious Disease Research, School of Life Sciences, Westlake University, Hangzhou 310024, Zhejiang Province, China

[4] Center for Future Foods, Muyuan Laboratory, Zhengzhou 450016, Henan Province, China

[*]**Corresponding author contact:** Tel.: 571-87963205 (lab), 571-87380995 (office), E-mail: jufeng@westlake.edu.cn

Huizhen Yan and Yunhua Zhang contributed equally to this work.

1. Supplementary figures and tables

Figure S1. The map of sampling stations colored by climate zone.

Figure S2. Rarefaction curves of species richness.

Figure S3. Spatial variation in microbial alpha-diversity and community composition.

Figure S4. Spearman correlation heatmap of α-diversity and dominant taxa with environmental factors.

Figure S5. Relationships between prokaryotic community βNTI and differences in key environmental factors.

Figure S6. Breakpoint analysis and community βNTI variation.

Figure S7. The relationships between microbial community assembly and Nitrososphaeria.

Figure S8. Co-occurrence network between Nitrososphaeria OTUs and dominant bacterial taxa.

Table S1. ANOVA analysis of prokaryotic community α-diversity across different vegetation types.

Table S2. The influence of environmental factors on surface soil microbial communities assessed by distance-based redundancy analysis (dbRDA).

Table S3. Mantel tests assessing Spearman correlations between surface soil microbial community βNTI and environmental factors across different vegetation types.

2. Supplementary Data Set

Dataset S1 The sampling information of the 205 samples across 125 sampling sites of the mainland China.

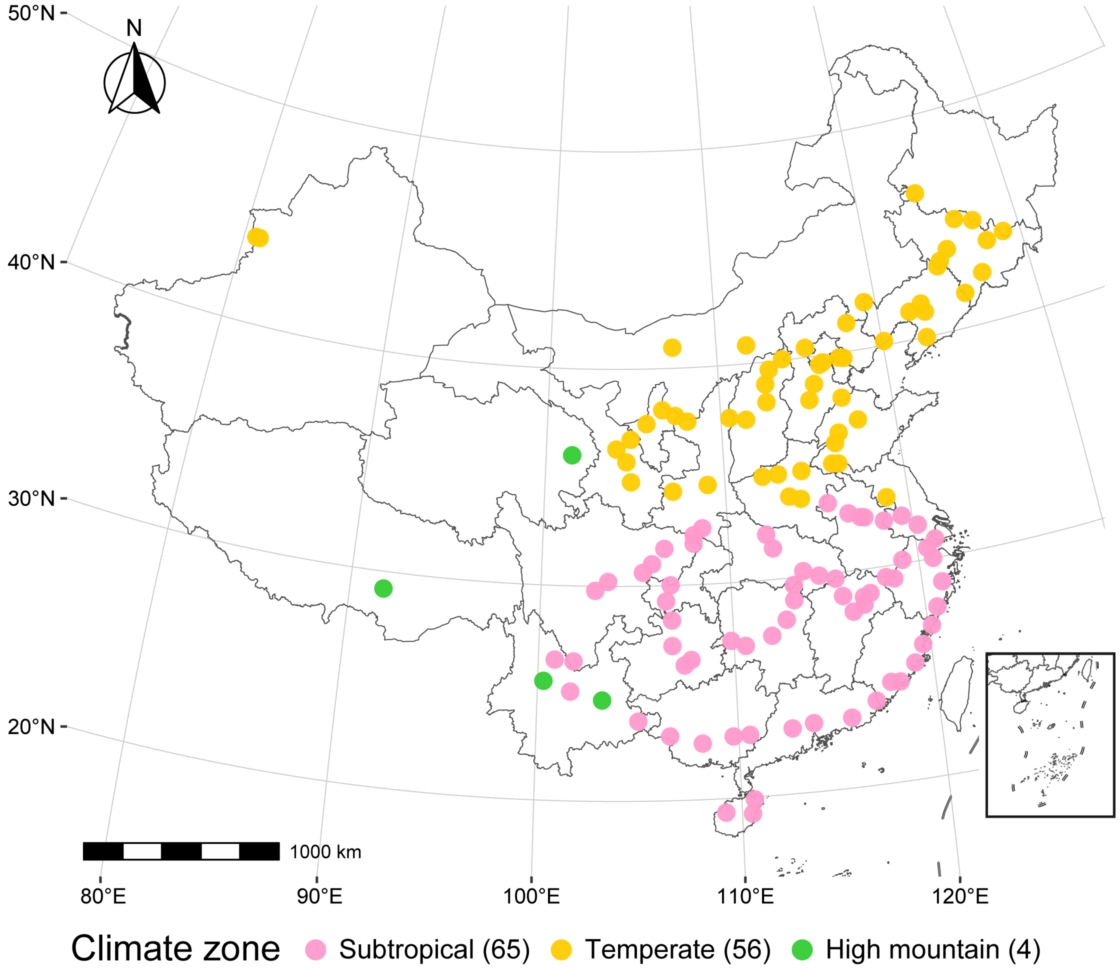

Figure S1. The map of sampling stations colored by climate zone (n = 125), The values in parentheses indicate the number of stations.

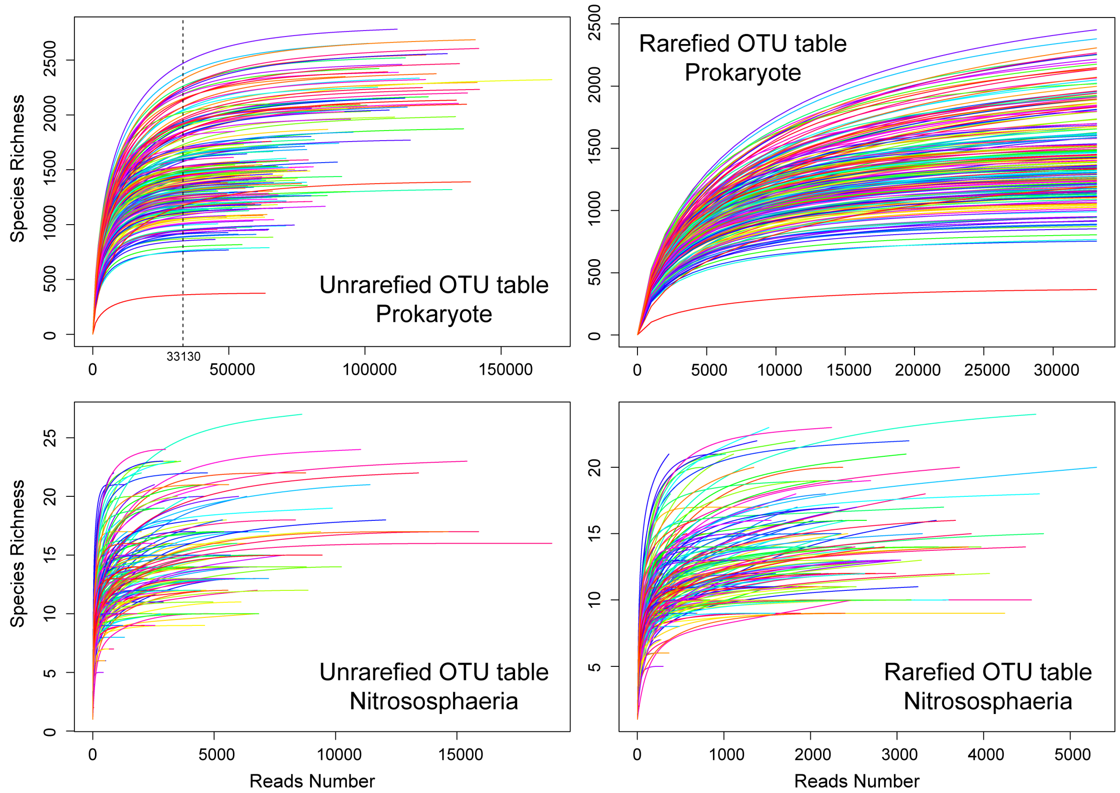

Figure S2. Rarefaction curves of species richness for prokaryotic communities and Nitrososphaeria based on rarefied and unrarefied OTU tables.

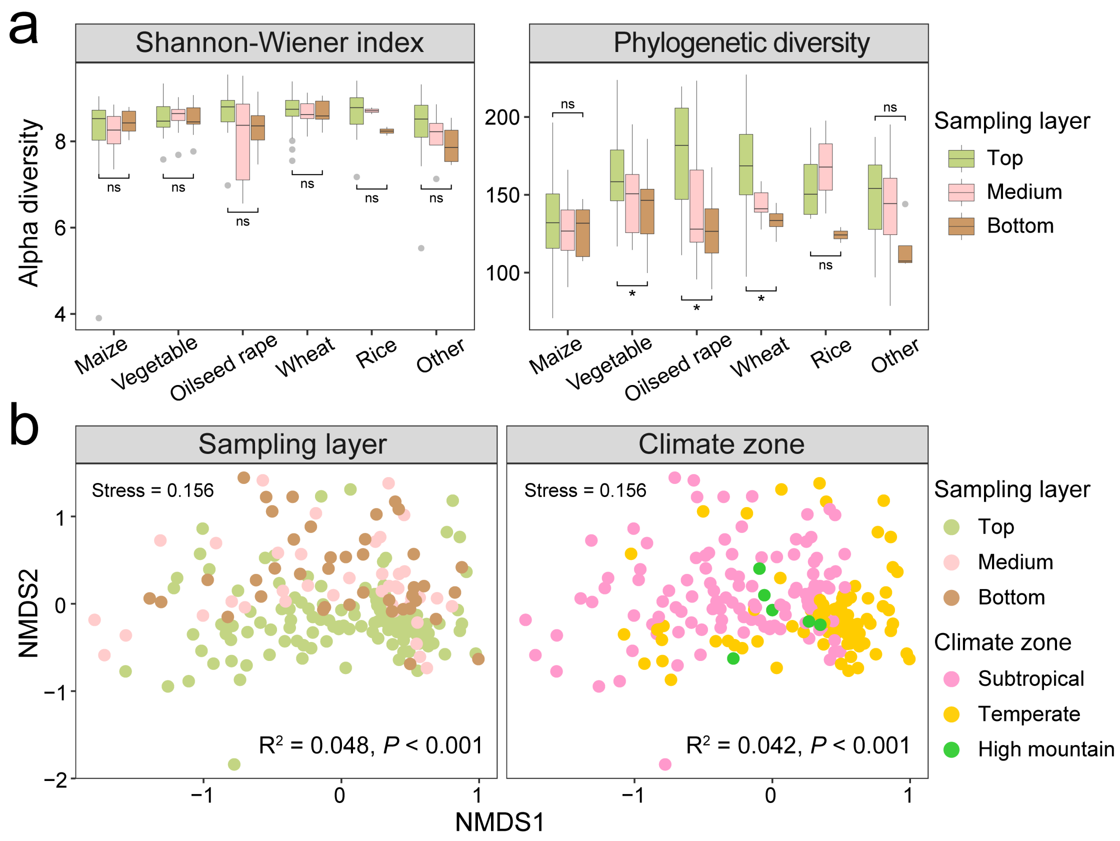

Figure S3. a: Spatial variation in prokaryotic alpha-diversity across vegetation types and sampling layers (n = 205). Differences in α-diversity across sampling depths were tested by ANOVA: ns = not significant; $^{*}P < 0.05$, $^{**}P < 0.01$. **b:** Non-metric multidimensional scaling (NMDS) ordination based on Bray-Curtis dissimilarity illustrating the compositional variation of microbial communities across sampling layers and climate zones (n = 205).

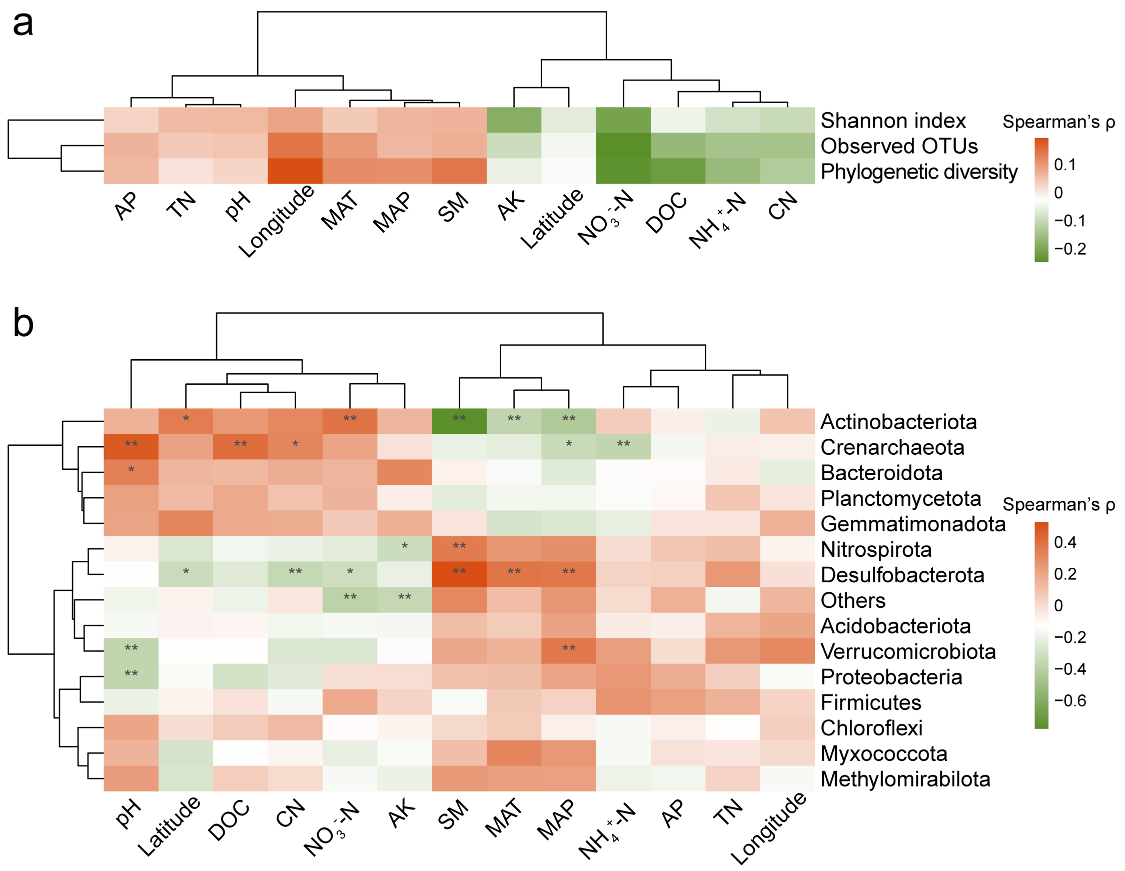

Figure S4. a: Spearman correlation heatmap between α-diversity of surface soil microbial communities and environmental factors (n = 125). **b:** Spearman correlation heatmap between the relative abundance of dominant microbial taxa in surface soil and environmental factors (n = 125). Holm-adjusted *P* values are indicated by asterisks in the cells: $^{*}P < 0.05$, $^{**}P < 0.01$. CN: carbon to nitrogen ratio; DOC: dissolved organic carbon; TN: total nitrogen; NO_3^--N: nitrate nitrogen; NH_4^+-N: ammonia nitrogen; AK: available potassium; AP: available phosphorus; SM: soil moisture; MAT: mean annual temperature; MAP: mean annual precipitation.

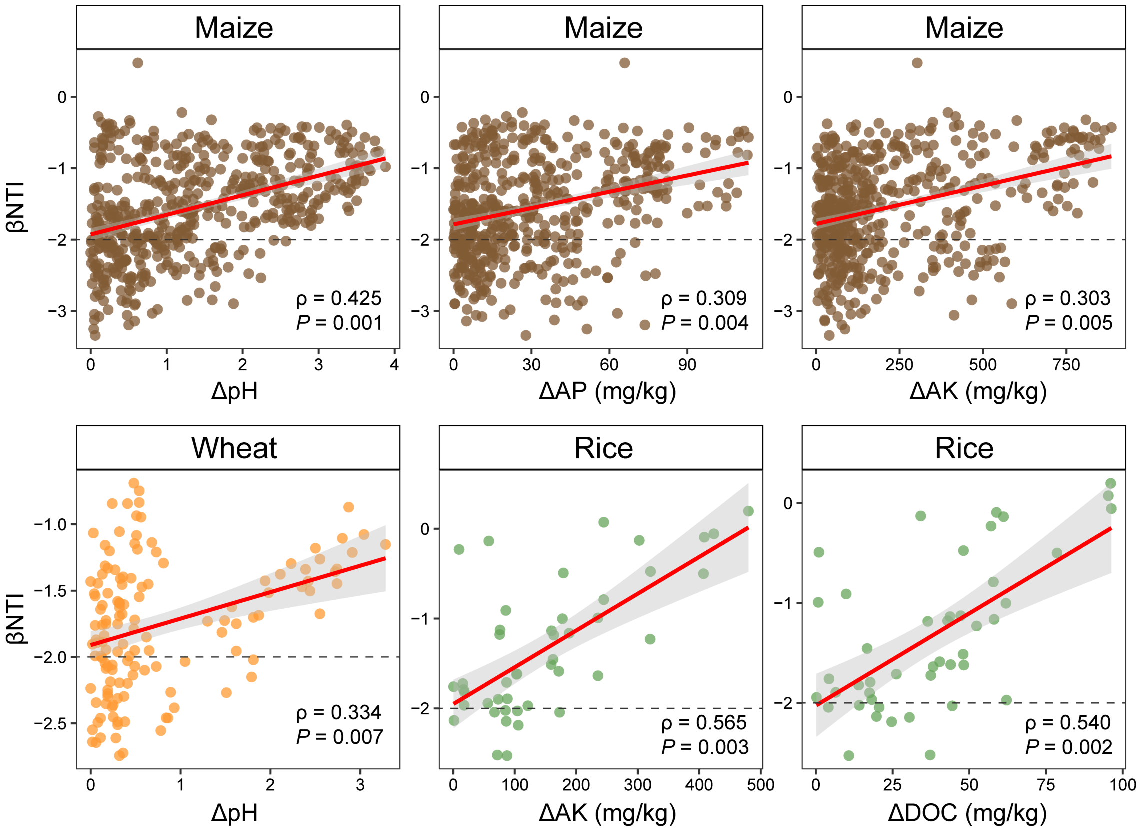

Figure S5. Relationships between prokaryotic community βNTI and differences in key environmental factors (Mantel test, Spearman's ρ > 0.3; refer to Table S3). Solid lines represent a linear fit. Horizontal dashed lines present thresholds at βNTI = -2.

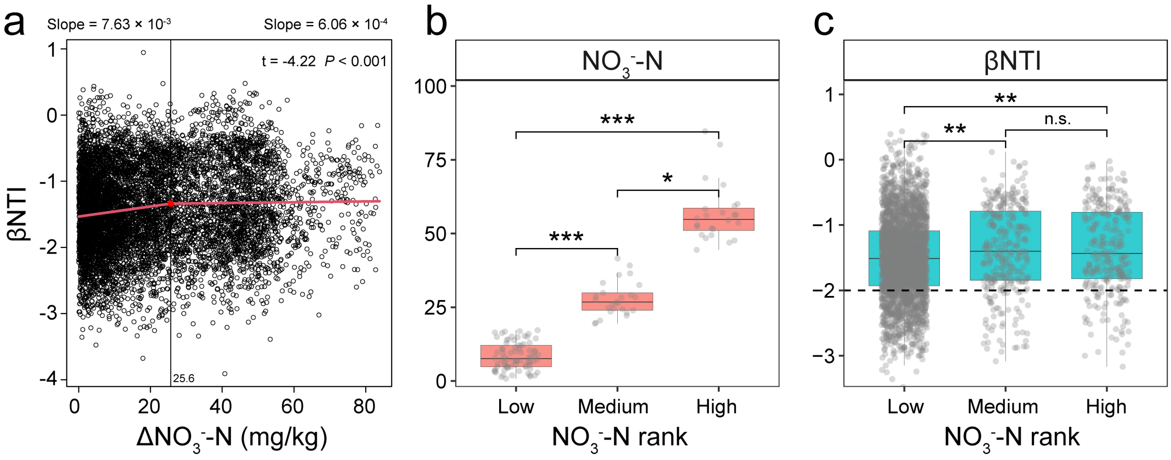

Figure S6. a: Breakpoint analysis of the relationship between βNTI and nitrate Euclidean distance. **b:** Gradient variation of nitrate. **c:** Changes in prokaryotic community βNTI along the nitrate gradient. *P*-values are marked with asterisks to indicate significance levels ($^{*}P < 0.05$, $^{**}P < 0.01$, $^{***}P < 0.001$, n.s. not significant) after Kruskal-Wallis Rank Sum Test.

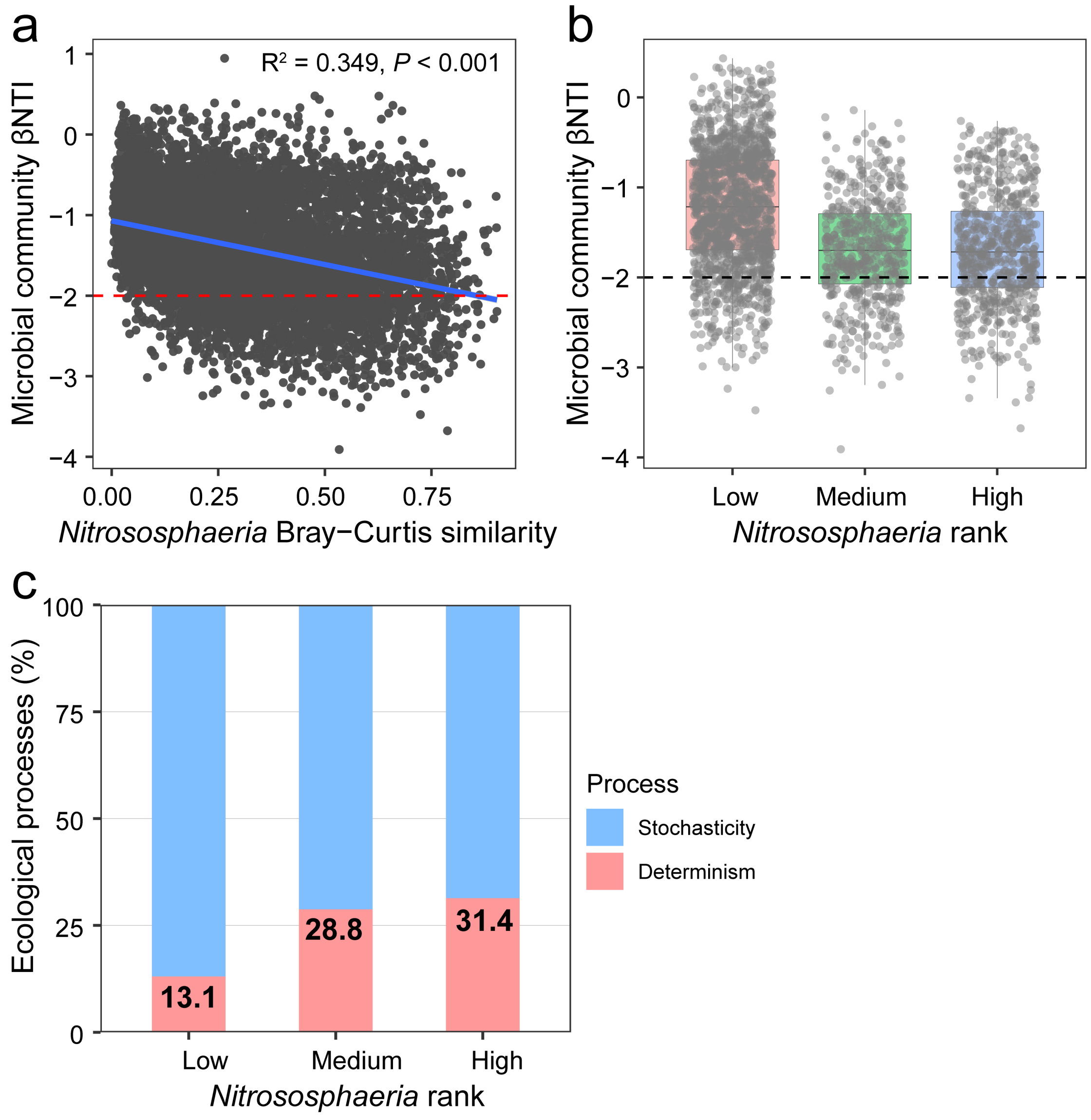

Figure S7. a: The relationships between microbial community βNTI values and Bray-Curtis similarity of Nitrososphaeria. **b:** Microbial βNTI distribution across different Nitrososphaeria abundance levels. **c:** Relative importance of assembly processes of microbial community using null models.

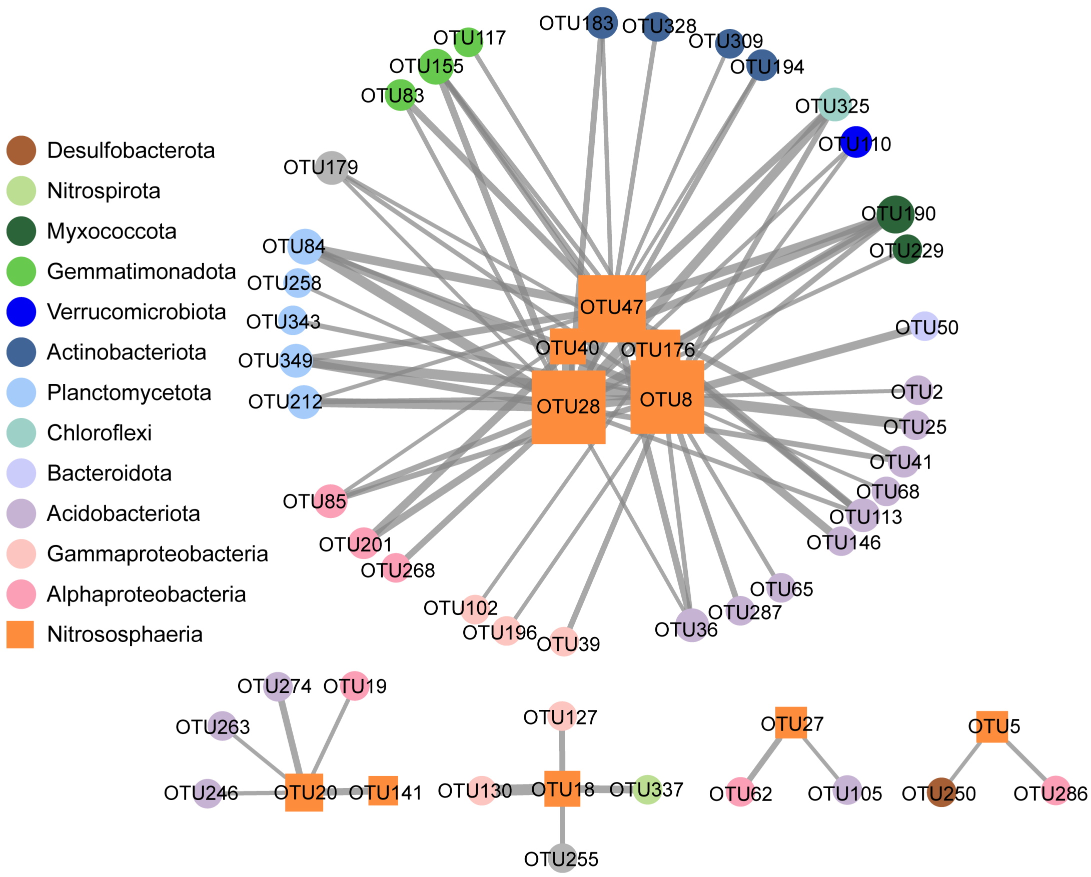

Figure S8. Co-occurrence network between Nitrososphaeria OTUs and dominant bacterial taxa. Orange squares represent Nitrososphaeria OTUs, while circular nodes are colored by dominant bacterial phyla or proteobacterial classes. Edges indicate significant positive correlations (Pearson's $|r| \geq 0.5$, $P < 0.05$), with line width proportional to the correlation strength.

Table S1. ANOVA analysis of prokaryotic community α-diversity across different vegetation types.

	Observed ASVs	Shannon-Wiener index	Phylogenetic diversity
All (n = 205)	**P < 0.001**	P = 0.063	**P < 0.001**
Top (0-15 cm) (n = 125)	**P < 0.001**	P = 0.049	**P < 0.001**
Medium (15-30 cm) (n = 40)	P = 0.750	P = 0.335	P = 0.556
Bottom (30-45 cm) (n = 40)	P = 0.423	P = 0.090	P = 0.388

Labels in bold indicate statistically significant differences at $P < 0.05$.

Table S2. The influence of environmental factors on surface soil microbial communities assessed by distance-based redundancy analysis (dbRDA).

**Categories**	**Factors**	**Explained variation (R^2)**	***P***
	pH	0.733	**< 0.001*****
	SM	0.452	**< 0.001*****
	DOC	0.277	**< 0.001*****
	CN	0.242	**< 0.001*****
Edaphic	NO_3^--N	0.198	**< 0.001*****
	AP	0.133	**< 0.001*****
	TN	0.108	**< 0.001*****
	NH_4^+-N	0.085	**0.004**
	AK	0.057	**0.036**
Climatic	MAP	0.535	**< 0.001*****
	MAT	0.291	**< 0.001*****
Geographic	Latitude	0.317	**< 0.001*****
	Longitude	0.086	**0.003**

P values marked with asterisks indicate environmental factors that significantly influence the variation in surface soil microbial communities (n = 125). $^{*}P < 0.05$, $^{**}P < 0.01$, $^{***}P < 0.001$. CN: carbon to nitrogen ratio; DOC: dissolved organic carbon; TN: total nitrogen; NO_3^--N: nitrate nitrogen; NH_4^+-N: ammonia nitrogen; AK: available potassium; AP: available phosphorus; SM: soil moisture; MAT: mean annual temperature; MAP: mean annual precipitation.

Table S3. Mantel tests assessing Spearman correlations between surface soil microbial community βNTI and environmental factors across different vegetation types.

	**Maize (n = 33)**		**Vegetable (n = 25)**		**Oilseed rape (n = 19)**		**Wheat (n = 17)**		**Rice (n = 10)**	
	**ρ**	***P***	**ρ**	***P***	**ρ**	***P***	**ρ**	***P***	**ρ**	***P***
pH	**0.425**	**0.001****	0.095	0.099	0.068	0.210	**0.334**	**0.007****	0.006	0.475
NO_3^--N	**0.242**	**0.011***	0.048	0.301	0.077	0.323	-0.031	0.592	0.409	0.058
MAP	**0.219**	**0.008****	**0.181**	**0.026***	0.054	0.309	**0.250**	**0.037***	0.226	0.063
AP	**0.309**	**0.004****	-0.046	0.668	-0.144	0.875	0.117	0.169	0.198	0.209
Latitude	0.087	0.134	**0.170**	**0.033***	0.018	0.419	0.061	0.266	0.089	0.284
AK	**0.303**	**0.005****	0.037	0.320	-0.146	0.867	0.059	0.303	**0.565**	**0.003****
Longitude	**0.112**	**0.041***	-0.111	0.940	**0.217**	**0.013***	0.067	0.303	-0.139	0.719
MAT	0.111	0.097	0.081	0.196	-0.145	0.857	0.103	0.203	0.128	0.246
NH_4^+-N	0.128	0.069	0.008	0.455	-0.217	0.936	0.171	0.104	0.370	0.052
SM	-0.009	0.523	0.041	0.337	**0.245**	**0.049***	0.013	0.448	-0.059	0.534
CN	0.072	0.214	0.114	0.173	0.137	0.173	0.108	0.155	0.037	0.396
DOC	0.025	0.403	-0.045	0.625	0.127	0.106	0.079	0.232	**0.540**	**0.002****
TN	-0.082	0.804	-0.102	0.869	-0.042	0.576	-0.090	0.776	-0.118	0.671

P-values are marked with asterisks to indicate significance levels ($^*P < 0.05$, $^{**}P < 0.01$). The 'n' in parentheses denotes sample size. CN: carbon to nitrogen ratio; DOC: dissolved organic carbon; TN: total nitrogen; NO_3^--N: nitrate; NH_4^+-N: ammonia; AK: available potassium; AP: available phosphorus; SM: soil moisture; MAT: mean annual temperature; MAP: mean annual precipitation.
